# Molecular Characterization and Expression of **α**-Globin and **β**-Globin Genes in the Euryhaline Flounder *(Platichthys flesus)*

**DOI:** 10.1155/2011/965153

**Published:** 2011-09-27

**Authors:** Weiqun Lu, Aurelie Mayolle, Guoqiang Cui, Lei Luo, Richard J. Balment

**Affiliations:** ^1^Key Laboratory of Exploration and Utilization of Aquatic Genetic Resources, Shanghai Ocean University, Shanghai 201306, China; ^2^Laboratory of Adaptive Biology, College of Fisheries and Life Science, Shanghai Ocean University, 999 Huchenghuan Road, Shanghai 201306, China; ^3^Faculty of Life Sciences, The University of Manchester, Oxford Road, Manchester M13 9PT, UK

## Abstract

In order to understand the possible role of globin genes in fish salinity adaptation, we report the molecular characterization and expression of all four subunits of haemoglobin, and their response to salinity challenge in flounder. The entire open reading frames of **α**1-globin and **α**2-globin genes were 432 and 435 bp long, respectively, whereas the **β**1-globin and **β**2-globin genes were both 447 bp. Although the head kidney (pronephros) is the predicted major site of haematopoiesis, real-time PCR revealed that expression of **α**-globin and **β**-globin in kidney (mesonephros) was 1.5 times higher than in head kidney. Notably, the **α**1-globin and **β**1-globin mRNA expression was higher than **α**2-globin and **β**2-globin in kidney. Expression levels of all four globin subunits were higher in freshwater- (FW-) than in seawater- (SW-)adapted fish kidney. If globins do play a role in salinity adaptation, this is likely to be more important in combating the hemodilution faced by fish in FW than the dehydration and salt loading which occur in SW.

## 1. Introduction

Haemoglobin (Hb) plays an important role in oxygen transport. In primitive vertebrates such as lamprey, haemoglobin is a monomer. However, in most vertebrate species, including teleost fish, haemoglobin is a tetrameric molecule that consists of two *α*-globin subunits and two *β*-globin subunits with one heme group. In human, the haematopoiesis process takes place in bone marrow, but teleost fish do not have bone marrow, and a number of studies have revealed that haemopoietic tissue is found mainly in the head kidney (pronephros) and a smaller amount in the kidney (mesonephros) [1, 2]. However, blood element formation differs among teleost fish [1, 3–5], and while previous studies indicated that head kidney, kidney, and spleen are the main organs forming blood in some species, erythropoiesis is mostly found in kidney [6, 7]. 

Fish can adequately supply oxygen to all tissues during environmental variations, such as temperature, pH, and oxygen tension changes, by increasing their total haemoglobin content or changing the intrinsic oxygenation characteristics [8, 9]. The haemoglobin-oxygen affinity is higher in hypoxic water than in oxygenated water [10, 11]; as pH decreases, the oxygen affinity of haemoglobin decreases [12, 13]. In order to increase total haemoglobin content, fish usually change globin gene expression at the site of haematopoiesis, which is in turn regulated by adaptive molecular mechanisms [14–16].

Most marine elasmobranchs transferred to lower salinity water exhibit marked changes in plasma and erythrocyte osmolyte composition [17–21]. Plasma dilution and loss of osmolytes can alter the oxygen-carrying capacity and haemoglobin oxygen-binding properties of the blood [22]. In *S. Aurata*, under low-salt concentration, *α*1 and *α*2 globin mRNAs levels of the red blood cells (RBCs) increased and decreased, respectively, compared with normal conditions [14]. Unlike marine elasmobranchs, euryhaline flounders are able to survive in both FW and SW but, in common with other euryhaline fish species, maintain a lower blood tonicity in FW. This reflects the long-term adjustments in metabolism and the profile of ion and water transport across gut, kidney, bladder, and gill epithelia that fish require to survive in hypotonic environments. In FW, fish maintain volume regulation by excreting large quantities of urine through increased renal filtration rate and renal tubule diameter to enhance urine flow [23]. The changing metabolic status and blood tonicity may also cause changes in oxygen consumption. In addition, a number of studies have revealed that nitrite, which is a potent vasodilator in humans, is bioactivated by reaction with deoxyhaemoglobin to preferentially generate nitric oxide (NO) under hypoxic conditions. The physiological function of deoxyhaemoglobin in this process is as an electronically and allosterically regulated nitrite reductase [24–27]. 

Here, we report the characterization of cDNAs encoding four types of globin in the flounder, along with a preliminary analysis of gene expression and tissue distribution of globins. The kidney was confirmed as the major site of globin expression in this species. Using quantitative real-time PCR measures of the four types of globin mRNA expression, we have examined differences in globin expression along with NO content in kidneys of chronically FW- and SW-adapted fish.

## 2. Materials and Methods

### 2.1. Animals

The flounder, *Platichthys flesus*, were collected from Morecambe Bay (Cumbria, UK) and transported to aquarium facilities at the University of Manchester. Flounder were of mixed sex and ranged in weight from 300 to 500 g. The flounder were maintained in recirculating, filtered SW (seawater, Natureland, Skegness, UK) or FW (tap water) tanks at 10–12°C under a 12 h : 12 h/light : dark photoperiod for at least 2 weeks before experimentation. All experiments were performed in accordance with United Kingdom Home Office Regulatory requirements.

### 2.2. Animal Experiments

To determine the steady-state conditions of fully acclimated animals, fish were studied after being held in SW or FW for 2 weeks in October. Fish were removed from tanks and without anesthetic, blood samples (3–6 mL) were collected within 90 sec into ammonium-heparinized syringes by needle puncture of the caudal blood vessels. Red blood cell count (RBC), hemoglobin (Hb), and hematocrit (Hct) were measured immediately, while plasma obtained by centrifugation of blood was analysed for total NO and electrolytes. The flounder were humanely killed and to reduce RBC contamination of tissue globin content blood was quickly removed from tissues by whole body perfusion via the heart with 50 mL ammonium-heparinized saline solution. Tissues (brain, spinal cord, CNSS, gill, head kidney, kidney, bladder, stomach, intestine, rectum, heart, spleen, liver, gonad, and muscle) were then removed and immediately snap frozen in liquid nitrogen. 

### 2.3. RNA Preparation

Total RNA was extracted from tissues using TRIZOL reagent in accordance with the manufacturer's protocol (Invitrogen, Paisley, UK), and RNA yield was quantified using a NanoDrop spectrophotometer ND-1000 (NanoDrop, Wilmington, DE). At this stage, total RNA from each sample (*n* = 8) was equally pooled for library construction and tissue distribution analysis. Before reverse transcription, total RNA was treated with deoxyribonuclease I (Invitrogen, UK) according to manufacturer's instructions. For the library, mRNA was purified from kidney total RNA using Dynabeads mRNA Direct kit (Dynal, UK).

### 2.4. cDNA Library Construction and EST Sequence

First strand cDNA synthesis was carried out using the Clontech SMART cDNA Library Kit (Clontech, Basingstoke, UK) as detailed by the manufacturers and amplified by polymerase chain reaction (PCR). The resulting cDNA was purified, cloned to the pDNR-LIB vector (Clontech, Basingstoke, UK), and then transformed into DH5*α* *Escherichia coli* (Eurogentec). Following cloning, 2,794 colonies (29 plates of 96 wells) were randomly picked from kidney library, sequenced (Macrogen, Seoul, Korea), and aligned with BLAST (basic local alignment search tool) against available databases using Trace2dBest [28]. The sequence alignment and homology analysis was performed using DNAMAN V4.15 software.

### 2.5. Northern Blot Analysis

10 *μ*g of total RNA from 15 perfused SW flounder tissues (brain, spinal cord, CNSS, gill, head kidney, kidney, bladder, stomach, intestine, rectum, heart, spleen, liver, gonad, and muscle) were electrophoresed on 1% denaturing agarose formaldehyde gel for 2.5 h at 150 V. The RNA samples were then blotted and subsequently fixed onto Hybond N nylon membranes (Amersham Biosciences, Buckinghamshire, UK) as previously described [29]. The full-length flounder *α*1-globin and *β*1-globin were used as cDNA probes for Northern blotting.

### 2.6. Real-Time Quantitative PCR Analysis

The tissue distribution of *α*-globin and *β*-globin mRNAs was analyzed by quantitative PCR of the 15 pooled total RNA tissue samples described above. The effect of salinity on kidney *α*-globin and *β*-globin mRNA expression was also examined for individual flounder samples (*n* = 7). All primers and TaqMan probes were designed using Primer Express (ABI) and synthesized commercially (Eurogentec, Seraing, Belgium), and the sequences are given in Table 1.

The optimization and validation of primers and probes were performed using standard ABI protocols. PCRs were performed in triplicate as described by Lu et al. [30]. For the relative quantification of *α*-globin and *β*-globin genes expression, the 2^−ΔΔCt^ method as fold changes in the target gene normalized to the reference gene and related to the expression of control was used. The internal control gene used for these analyses was the housekeeping gene 18S, though comparable results were also obtained with *β*-actin. 

### 2.7. *In Situ * Hybridisation (ISH)

The head kidney and kidney were dissected and fixed in 4% paraformaldehyde [31] and embedded in paraffin wax. Longitudinal 4 *μ*m thick sections were cut, mounted on positive charged slides, and incubated at 60°C for 5 days. *In situ *hybridization was carried out as previously described [29]. The full-length flounder *α*1-, *α*2-, *β*1-, and *β*2-globin were used to synthesize ^35^S-labelled RNA probes for *in situ* hybridisation.

### 2.8. Immunocytochemistry

The *α*-globin (H80) or *β*-globin (H-76) antibodies were obtained from Santa Cruz Biotechnology Inc. (Santa Cruz, Calif). These rabbit polyclonal antibodies were raised against amino acid 62–142 of human haemoglobin *α* and amino acid 62–147 mapping near the C-terminus of human haemoglobin *β*. Antibody specificity was tested by Western blotting on Wistar rat and flounder blood cell samples. The proliferating cell nuclear antigen (PCNA, C-20; Santa Cruz) antibody is a goat polyclonal IgG mapped at the C-terminus of PCNA of human origin. PCNA is a highly conserved protein in both animal and plant systems and was used as a marker for the heamopoietic stroma of kidney. Immunocytochemistry was carried out based on the method of Lu et al. [30] using goat antirabbit or rabbit antigoat antiserum (DAKO, UK), respectively, as the linking reagent and diaminobenzidine as the chromogen. Control experiments were carried out by omitting the primary antibody.

### 2.9. Total NO Measurement

To assess the relative nitric oxide status of SW and FW fish kidneys, renal tissue samples from 7 SW- or 7 FW-adapted fish were pooled. NO breakdown products (NO*_x_*) were measured using an enzymatic test (Immundiagnostik, Bensheim, Germany) according to the manufacturer's instructions. 

### 2.10. Statistical Analysis

Plasma and blood analysis data are presented as means ± SE, and comparisons are by *t*-test. Results for tissue measurements of the mRNA levels of *α*1-globin, *α*2-globin, *β*1-globin, and *β*2-globin are also expressed as means ± SE. Differences between groups were analysed by ANOVA. Significance levels were set at *P < 0.05.

## 3. Results

### 3.1. Isolation and Characterization *α*-Globin and *β*-Globin cDNA

**Figure 1:**
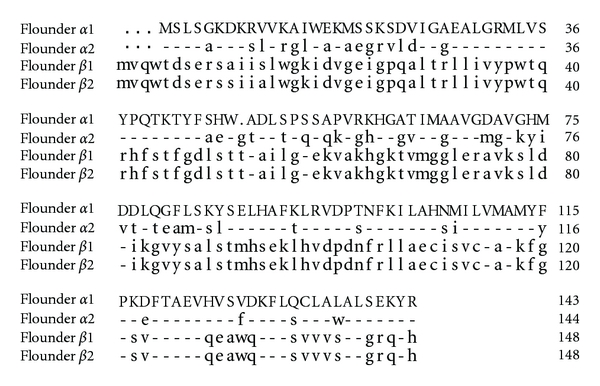
Alignment of deduced amino acid sequences of two types of the flounder *α*-globin cDNAs and two types of the flounder *β*-globin cDNAs. Dot indicates that the amino acid is absent. Dash indicates the identical amino acid.

Six *α*1-globin (accession number: HQ843790), three *α*2-globin (Accession number: HQ843791), six *β*1-globin (accession number: HQ843792), and two *β*2-globin (accession number: HQ843793) cDNA clones were obtained following sequence screening of 2,794 randomly picked colonies from the kidney library. They were characterized by nucleotide sequence analysis. The entire open reading frames (including stop codon) of *α*1 and *α*2 genes were 432 and 435 bp long, encoding a putative protein of 143 and 144 amino acids, respectively. The *α*2-globin had a Gly inserted at residue 48 (Figure 1). Comparison of the deduced amino acid sequences of flounder *α*1-globin and human *α*-globin polypeptide revealed that 12 of 16 heme interfaces, 9 of 16 *α*1*β*1 interfaces and 11 of 14 *α*1*β*2 interfaces were similar, but 5 of 6 Bohr effect residues were different (Supplementary, see Figure 1(a) in Supplementary Material available online at doi: 10.1155/2011/965153). The homology between *α*1 and *α*2 was 58.3% for nucleotides and 65.7% for deduced amino acids. The open reading frames (including stop codon) of *β*1 and *β*2 genes were both 447 bp encoding a putative protein of 148 amino acids. The homology between *β*1 and *β*2 was 89.9% for nucleotides and 98.6% for deduced amino acids. In the flounder *β*1-globin, 11 of 16 heme interfaces, 9 of 16 *α*1*β*1 interfaces, and 11 of 13 *α*1*β*2 interfaces were similar to those in human *β*-globin (Supplementary, see Figure 1(b) in Supplementary Material available online at doi: 10.1155/2011/965153). The deduced amino acid sequences of *α*-globin and *β*-globin only shared 15% identity. 

### 3.2. Alignment of *α*-Globin and *β*-Globin Amino Acid Sequences

**Figure 2:**
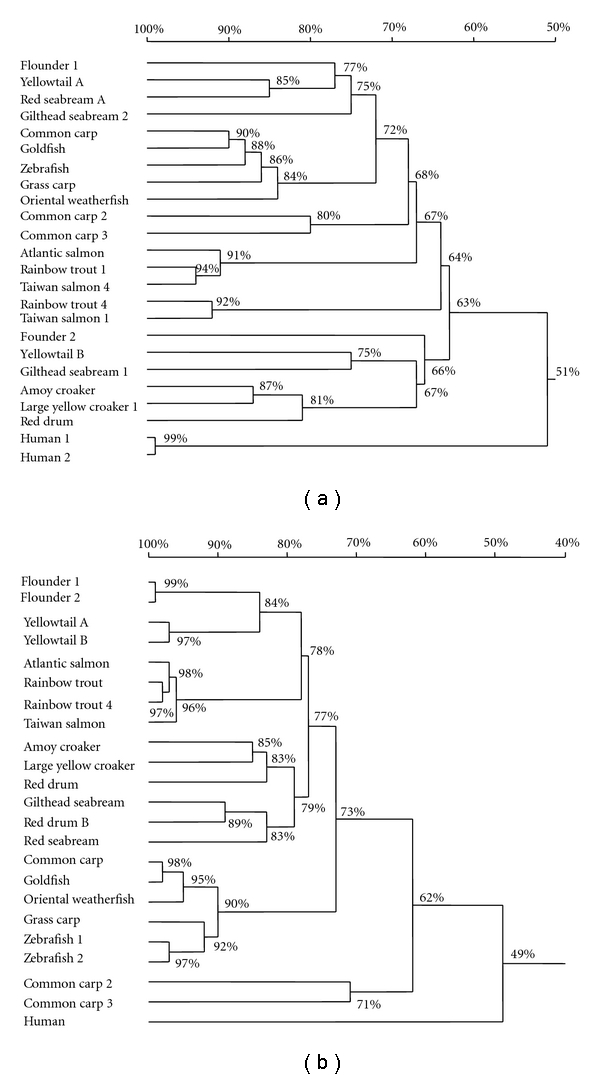
Homology tree of flounder *α*-globin and *β*-globin. (a) Comparison of the predicted amino acid sequences of the flounder *α*-globin with corresponding chains from previously known fish and human *α*-globin. DDBJ accession numbers: red drum (AAX35759); Amoy croaker (AAZ79649); large yellow croaker 1 (AAV52697); yellowtail A (BAA86218); yellowtail B (BAA86219); gilthead seabream 1 (ABF67512); gilthead seabream 2 (ABF67513); goldfish (CAP69820); grass carp (AAM93257); common carp (BAA20511); common carp 2 (BAB79237); common carp 3 (BAB79240); oriental weatherfish (AAM93258); taiwan salmon 1 (ABY21328); taiwan salmon 4 (ABY21327); red seabream A (AAP20155); atlantic salmon (CAA65949); zebrafish (NP_571332); rainbow trout 1 (NP_001118022); rainbow trout 4 (NP_001118023); human 1 (AAK37554); human 2 (AAN04486). (b) Comparison of the predicted amino acid sequences of the flounder *β*-globin with corresponding chains from previously known fish and human *β*-globin. DDBJ Accession Numbers: yellowtail A (BAA86220); yellowtail B (BAA86221); goldfish (CAP69821); grass carp (AAM93253); common carp 1 (BAA13536); common carp 2 (BAB79238); common carp 3 (BAB79239); zebrafish 1 (AAI15159); zebrafish 2 (AAH53176); large yellow croaker (AAV91971); oriental weatherfish (AAM93260); amoy croaker (AAZ79648); taiwan salmon (ABY21329); rainbow trout 1 (ACO07479); rainbow trout 4 (ACO08017); red seabream (AAP20173); atlantic salmon (ACI68343); red drum A (AAW55624); red drum B (AAZ14832); gilthead seabream (ABE2802); human (ACU56984).

Cluster analysis of the two types of flounder *α*-globin cDNAs named *α*1 and *α*2 is presented in Figure 2(a). The homology of flounder *α*1 and *α*2 was 65.7%, and they were placed in different homology tree clusters. Amino acid homology of the flounder *α*1-globin gene compared with other reported fish ranged from 62.2% to 79.7%, whereas homology for *α*2-globin amino acid sequences was between 52.4% and 68.1%. The flounder *α*1-globin shared 50.0% and *α*2-globin 43.7% sequence homology with human *α*1- and *α*2-globin. Irrespective of the different nomenclature, the flounder *α*1-globin was closely related to yellowtail *α*-globin type A, red seabream *α*-globin type A, and gilthead seabream *α*-globin type 2, whereas flounder *α*2-globin was more closely related to yellowtail *α*-globin type B, Amoy croaker *α*-globin type 2, large yellow croaker *α*-globin type 1, red rum *α*-globin type 2, and gilthead seabream *α*-globin type 1.

The homology analysis of the flounder *β*-globin cDNAs is shown in Figure 2(b), both have 148 amino acid residues. *β*1 and *β*2 shared 98.6% sequence homology and were placed together in the same homology tree cluster. Comparison of flounder *β*-globin with orthologous vertebrate *β*-globin showed that flounder *β*-globin shared between 59.9% and 85.0% homology with other fish *β*-globin, and only 50.3% homology with human *β*-globin. Cluster analysis showed that the *β*-globin gene in yellowtail was closest to that in flounder.

### 3.3. Tissue Distribution of Flounder *α*-Globin and *β*-Globin mRNA

**Figure 3:**
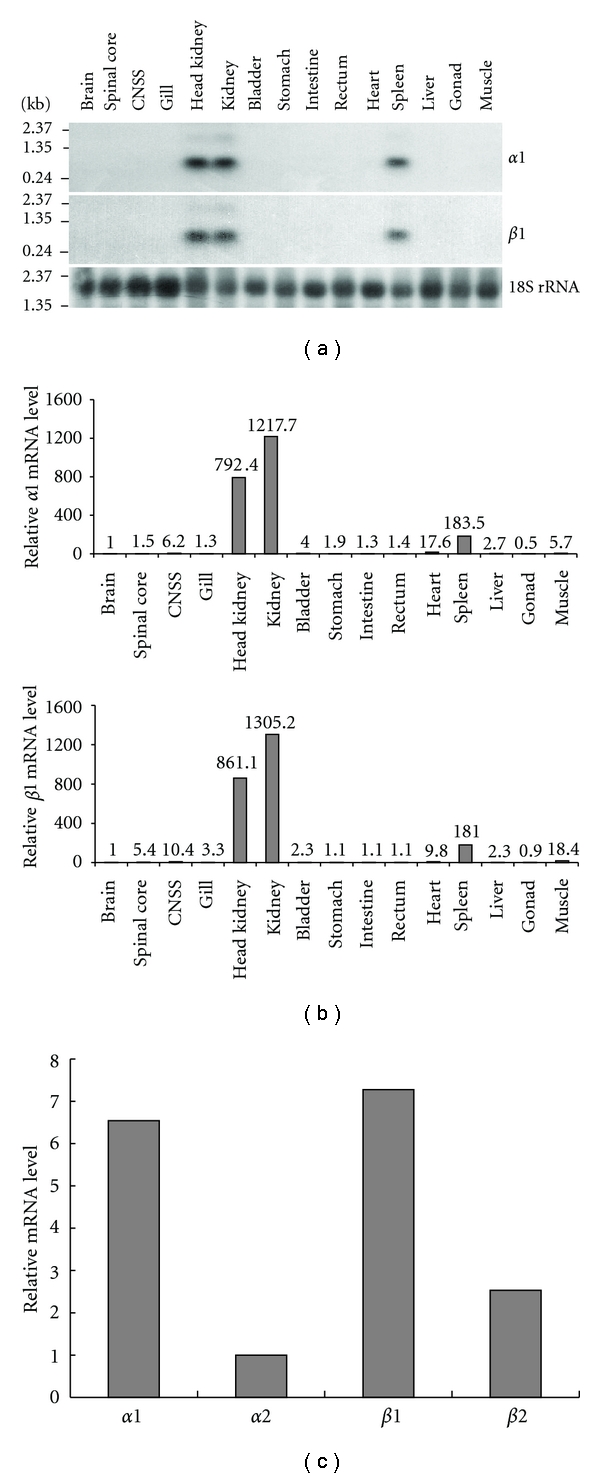
Tissue distribution of *α*-globin and *β*-globin mRNA. (a) Northern blot showing tissue distribution and size of the *P. flesusα*1-globin and *β*1-globin transcripts. (b) Relative mRNA expression levels of *α*1-globin and *β*1-globin in different tissues. *α*-globin and *β*-globin expressions in different tissues were analyzed by real-time qPCR with 18S rRNA as reference gene. Values are relative fold change with brain as 1 for pooled samples from 8 SW-acclimated adult flounder; (c) expression levels of four types of globin mRNAs in kidney. *α*-globin and *β*-globin expressions in kidney were analyzed by real-time qPCR with 18S rRNA as reference gene. Values are relative fold change with *α*2-globin as 1 for pooled samples from 8 SW-acclimated adult flounder.

Northern analysis on a range of flounder tissues showed that *α*-globin and *β*-globin transcripts were only present in head kidney, kidney, and spleen RNA samples (Figure 3(a)). The major band size was approximately 650 nucleotides, which was consistent with the predicted length based on the cDNA clones obtained. The relative *α*-globin and *β*-globin mRNA expression levels in different tissues were also determined by real-time PCR (Figure 3(b)). The expression levels of *α*-globin and *β*-globin mRNA in head kidney and kidney were much greater than in other tissues, while the spleen also appeared to express relatively high levels of *α*-globin and *β*-globin transcripts. The expression level of *α*-globin and *β*-globin mRNA in the kidney was 1.5 times higher than those in head kidney. In kidney, expression levels of the four individual types of globin mRNA also differed.  Figure 3(c) shows that the expression levels of *α*1-globin and *β*1-globin mRNAs were higher than those of *α*2-globin and *β*2-globin mRNAs.

### 3.4. Differential Expression of Globins between SW- and FW-Adapted Fish

**Figure 4:**
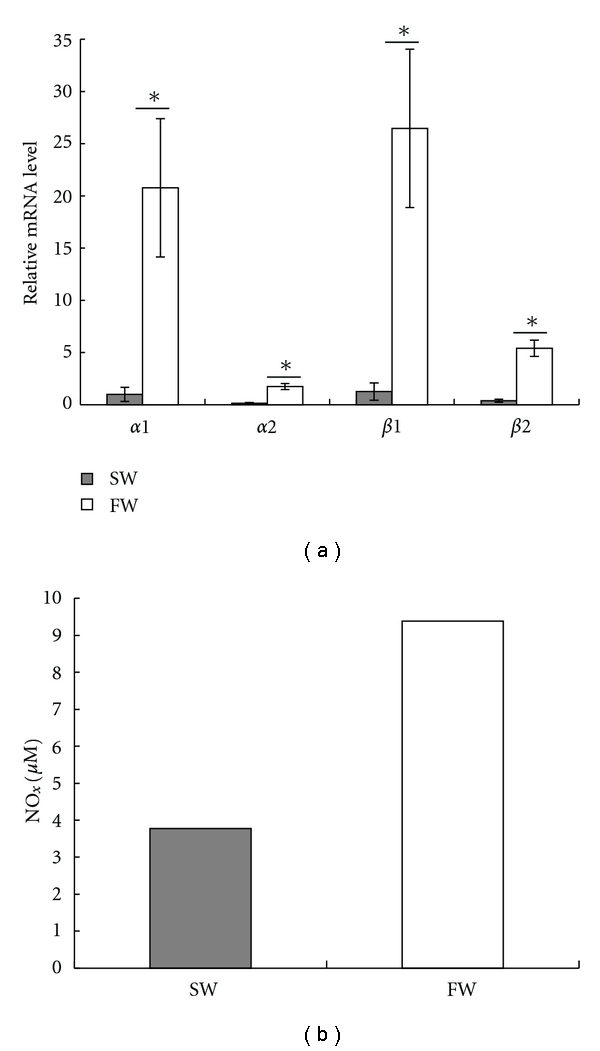
Relative mRNA expression levels of four types of globin and concentration of NO*_x_* in kidney of FW- and SW-adapted flounder. (a) Expressions of four types of globin were analyzed by real-time qPCR with *β*-actin as reference gene. Values are relative fold change with *α*2-globin in SW-adapted flounder kidney as 1; the significant difference between FW- and SW-adapted flounder was indicated as *P < 0.05 (*n* = 7). (b) Concentration of total NO in kidney. Values are measurements from 7 pooled kidneys of FW- or SW-adapted flounder.

Blood composition of chronically FW- and SW-adapted flounder is shown in Table 2. Plasma chloride, magnesium, and osmolality were significantly higher in SW- than in FW-adapted fish. No significant differences were evident for calcium, potassium, and sodium between long-term SW- and FW-adapted fish. There were also no significant differences in blood hematocrit, RBC, and haemoglobin content, which were slightly higher in FW- than in SW-adapted fish. Although there were no statistically significant differences in RBC or haemoglobin content between FW- and SW-adapted flounder, expression levels of *α*-globin and *β*-globin mRNAs in the kidney were significantly higher in FW- than in SW-adapted fish (Figure 4(a)). Notably, the pooled kidney NO*_x_* level in FW-adapted fish was double that in SW- adapted fish (Figure 4(b)). 

**Figure 5:**
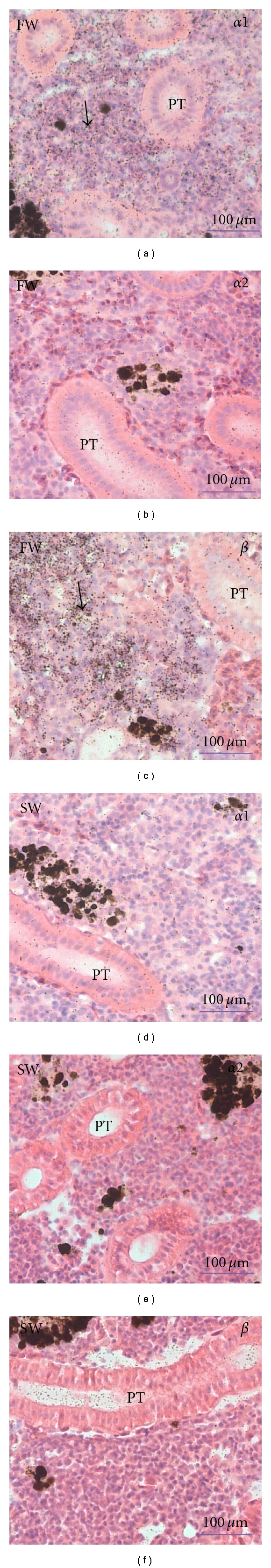
*In situ* hybridization for *α*1-globin, *α*2-globin, and *β*-globin in kidney of FW- and SW-adapted flounder. Abundant *α*1-globin and *β*-globin gene expression in haematopoietic stroma of FW-adapted flounder is shown with antisense *α*1-globin and *β*-globin ^35^S RNA probes, and *α*1-globin and *β*-globin mRNA was not detected in SW-adapted flounder. *α*2-globin mRNA was not detected in either of FW- and SW-adapted flounder kidney using an antisense *α*2-globin ^35^S RNA probe. The haematopoietic stroma with abundant globins expression are indicated by solid arrows. PT: proximal tubule.

Further study of the kidney as the major site of *α*-globin and *β*-globin expression, by *in situ* hybridisation using ^35^S-labeled *α*-globin and *β*-globin RNA probes (antisense) showed that all four types of globin genes were expressed in cells around the renal blood vessels (Figure 5). Abundant expression of both *α*1 and *β* globin (both types of *β* globin) genes was evident in the FW-adapted fish kidney, and the incidence and apparent density of signal was much higher for *β*-globin than *α*1-globin. There was no precise signal detected in *α*2-globin-hybridised tissue sections. In SW-adapted fish kidney, the signal obtained was weak, and no cells contained precisely dark dots. The transcriptional activity of both *α*- and *β*-globin was clearly higher in FW- than in SW-adapted fish kidney tissue sections. RBCs were void of signal. The negative controls showed few silver grains, indicating the background produced by weak nonspecific binding (Supplementary, see Figure 2 in Supplementary Material available online at doi: 10.1155/2011/965153). 

**Figure 6:**
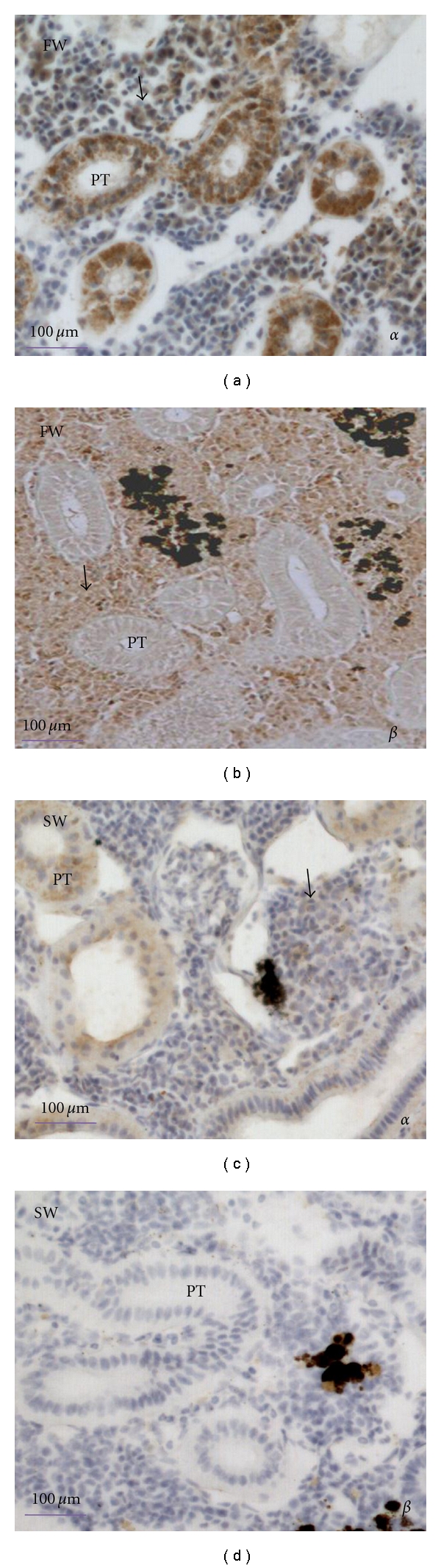
Immunocytochemistry for *α*-globin and *β*-globin in kidney of FW- and SW-adapted flounder. The immunoreactivity of *α*1-globin was located in both of external contour of tubules and haematopoietic stroma, whereas *β*-globin was localised only in haematopoietic stroma in FW-adapted fish kidney. The haematopoietic stroma with abundant globins is indicated by solid arrows. PT: proximal tubule.

**Figure 7:**
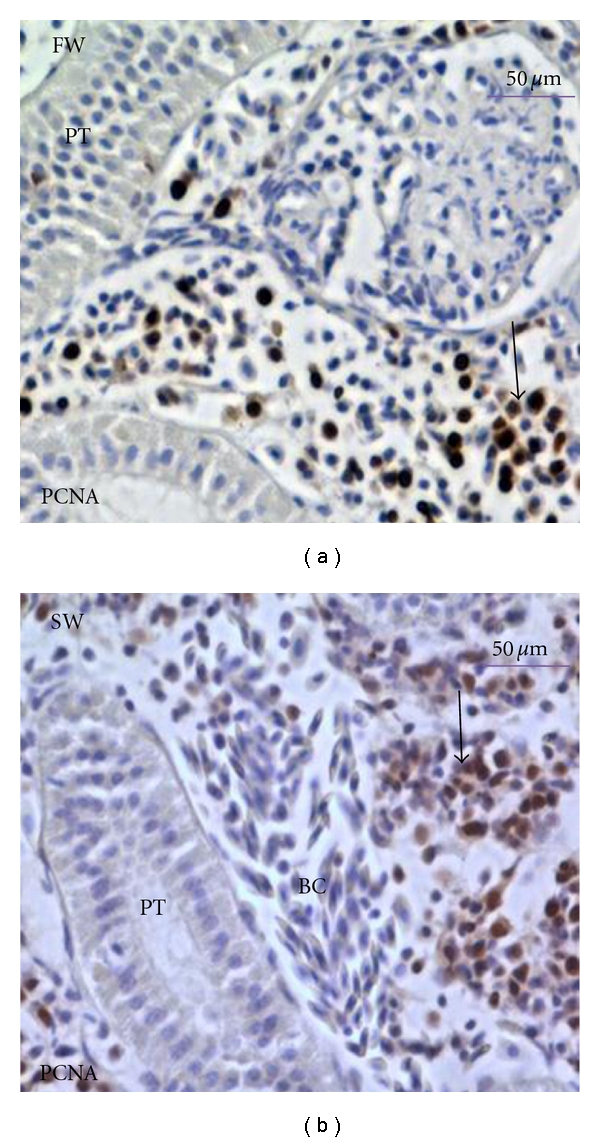
PCNA immunocytochemistry in kidney of FW- and SW-adapted flounder. The immunoreactivity of PCNA was located in haematopoietic stroma. The haematopoietic stroma with abundant PCNA is indicated by solid arrows. PT: proximal tubule; BC: blood cell.

In FW-adapted fish kidney, the immunoreactivity of *α*1-globin was located in both the haematopoietic stroma and the external contour of renal tubules, whereas *β*-globins were localised only in the haematopoietic stroma. In SW-adapted fish kidney, *α*1-globin was localised in the haematopoietic stroma and internal contour of tubules, but there was no detectable signal for *β*-globin (Figure 6). The protein expression level seemed to be higher in FW- than in SW-adapted kidney tissues. Immunoreactivity for PCNA in kidney appeared limited to the haematopoietic stroma, and again the expression level seemed to be higher in FW- than in SW-adapted kidney tissues (Figure 7). The heterogeneous nature of the results regarding the peroxidase precipitates intensity in kidney tissue sections and obviates more precise statements regarding the levels of PCNA, *α*-globin, and *β*-globin present.

## 4. Discussion

This paper is the first to describe the cloning and molecular characterization of flounder *α*- and *β*-globin genes, and to examine the effect of altered environmental salinity on *α*- and *β*-globin gene expression. 

### 4.1. Molecular Identification and Characterization of *α*-Globin and *β*-Globin cDNA

Two *α*-globin genes were isolated, and the homology of deduced amino acid sequences was 65.7%. The *α*2-globin had one Gly insertion at position 48. However, Yellowtail *α*-globin type B has a Gly insertion at position 47 [32, 33]. Miyata et al. [33] suggested that an insertion of Arg or Asp residue at position 47 in carp *α*-globin produced only inconsequential changes in function and the three-dimensional structure of globin. Despite these structural similarities, a comparison of deduced amino acid sequences of the two types of flounder *α*-globin placed them in different homology tree clusters, namely, those that are structurally more akin to *α*1-globin, and those more akin to *α*2-globin. Flounder *α*1-globin is most closely related to that of yellowtail *α*-globin type A, red seabream *α*-globin type A, and gilthead seabream *α*-globin type 2 (75–78%), while Salmonidae *α*1-globin only shares 68% identity with flounder and occupies a separate group. The other two groups consist of the rest of known freshwater teleosts, namely, carp, goldfish, and zebrafish (72–75%), and a separate group which contains human *α*-globin (Figure 2(a)). 

We isolated two *β*-globin genes in flounder, and the deduced amino acid sequences were 98.6% homologous, and considerably homologous with other vertebrate *β*-globins. Both types of flounder *β*-globin had a Phe residue at position 121, in contrast to a Lys residue in yellowtail *β*-globins, when compared with human *β*-globin [32]. Yoshizaki et al. [34] suggested that such a residue insertion may affect the function of *β*-globin. The homology tree was consistent with the phylogeny based on classical taxonomy. The two types of *β*-globin were placed together and although yellowtail *β*-globin was the closest *β*-globin of members of Sciaenidae, Cyprinidae, and Salmonidae were also close to that of flounder. It appears that *β*-globins are more conserved than *α*-globin in fishes. 

### 4.2. Tissue Distribution of Flounder Globin mRNAs

Northern blot analysis of total RNA prepared from a range of flounder tissues revealed that the kidney (mesonephros), head kidney (pronephros), and spleen are major sites of expression of the *α*-globin and *β*-globin transcripts. Previous histological studies in *Clarias gariepinus* and *Sarotherodon mossambicus* revealed that the kidney, head kidney, and the spleen are the main organs forming blood [6]. Boomker also demonstrated that the cells forming the erythroid and granuloid lineage are mostly found in kidney, while thrombocytes and monocytes are formed in head kidney and spleen in these species [7]. The presence of higher levels of *α*-globin and *β*-globin transcripts in kidney, suggests that the kidney may be a more important site of erythropoiesis than head kidney and spleen in flounder.

### 4.3. Differential Expression of Globins between SW and FW

European flounders are able to survive in both FW and SW but, in common with other euryhaline fish species, maintain a lower blood tonicity, and also increased urine volume and reduced urine osmolality in the FW environment. In terms of blood oxygen transport, we did not see significant differences in blood hematocrit, RBC, or haemoglobin content between FW- and SW-adapted fish. In contrast, the expression levels of all four types of globin mRNAs in the kidney were significantly higher in FW-adapted compared with SW-adapted fish. This is consistent with the observed expression changes of two *α*-globins following exposure of the elasmobranch, *S. aurata*, to low salinity [14]. These observations suggest that Hb may be involved in some aspects of salinity adaption in fish although exactly the mechanism by which Hb is employed remains unclear.

To clarify these important observations, further investigations were performed using histochemical methods. The apparent increased necessity for globin protein in FW-adapted fish was further supported by the higher globin mRNA expression determined by ISH. This increased mRNA expression in FW kidney was regionalised in the haematopoietic stroma, in a group of cells probably associated with the early stages of erythrocyte generation, but no expression was identified in RBCs themselves. This was coincident with apparently greater immunoreactivity of PCNA, a cell proliferation marker, in the haematopoietic stroma of FW kidney tissue. Importantly, the immunohistochemistry results also showed that both *α*- and *β*-globin subunits were located in haematopoietic stroma, confirming the kidney as a major site of production of all four types of globin and of the erythropoietic processes. 

In humans, deoxyhaemoglobin is an electronically and allosterically regulated nitrite reductase, and it can convert nitrite to NO preferentially under hypoxic conditions [24–27]. NO may also be controlled by haemoglobin binding properties [35]. Interestingly, kidney tubules dilate and tubule diameter increase in FW flounder [23]. The finding that kidney total NO level in FW-adapted fish was double that in SW-adapted fish, in combination with our previous studies [23], suggests that NO could perhaps play a role in variation of tubule diameter dependent upon haemoglobin binding and reductase activity. The exact mechanisms clearly warrant further investigation.

## 5. Conclusion

From this study, we conclude that head kidney, kidney, and spleen are erythropoietic tissues in flounder, The kidney is the major site of production of all four types of globin although all four types of globin genes are not expressed equally. Importantly, we also found that *α*- and *β*-globin mRNAs are differentially expressed in chronically FW- and SW-adapted flounder, which raises the possibility that, in fish, these globins may be important in some aspects of salinity adaptation. The newly generated deoxyhaemoglobin may play an important role in combating the hemodilution faced in FW. This may be particularly relevant for migratory fish such as flounder and salmonids as they move between media of different salinities. Similar mechanism may also be of importance in mammals and would be of interest to examine in the future.

## Supplementary Material

Supplementary Figure 1: Comparison of the predicted amino acid sequences of (a) the flounder **β**- globin with corresponding chains from previously known **α**-globin of yellowtail (Okamoto et al., 2001) and human (Michelson and Orkin, 1980) , and (b) the flounder **β**-globin with **β**-globin of yellowtail (Okamoto et al., 2001) and human (Lawn et al., 1980). The beginnings and ends of the helical regions, as established for mammalian globins, are indicated by (+) and (-), respectively. The positions of functional residues are indicated by: H, heme binding; 1, **α**1**β**1 interfaces; 2, **α**1**β**2 interfaces; and B, Bohr effect residues.
Supplementary Figure 2: Negative control for figure 5 *&* 6. (a) In situ hybridization. The same group of cells showed no specific signal when the specific antisence probe for **α**-globin and **β**-globin was replaced with sence probe. (b) Immunocytochemistry. The same group of cells showed no immunoreactivity when the specific primary antiserum for **α**-globin and **β**-globin was replaced with preimmune rabbit serum. PT, Proximal tubule.

## Figures and Tables

**Table 1:** Gene-specific primers and probes for *α*-globin, *β*-globin, *β*-actin, and 18S rRNA.

Name of primer	Sequence of primer 5′-3′
Hb*α*1 sense-221F	TCCGCTCCGGTGAGGAA
Hb*α*1 antisense-323R	GCTCGCTGTATTTGGAGAGGAA
Hb*α*1 TaqMan probe-273T	6-FAM-CCGTCGGACACATGGATGATCTTCAA-TAMRA
Hb*α*2 sense-261F	GGGCATGGGCGTGAAAT
Hb*α*2 antisense-329R	AAGGCGTGCAGCTCACTGA
Hb*α*2 TaqMan probe-279T	6-FAM-CATCGTTACTCTCACCGAAGCCAT-TAMRA
Hb*β*1 sense-F	CATGGTCCAGTGGACAGATAGT
Hb*β*1 antisense-R	CTCCCCCACATCGATTTTTC
Hb*β*1 TaqMan probe-T	6-FAM-AGCGCAGCGCCATCATTTCCCTTTG-TAMRA
Hb*β*2 sense-30F	CATGGTCCAGTGGACAGATAGC
Hb*β*2 antisense-100R	TCTCCCCCACATCGATTTTC
Hb*β*2 TaqMan probe-53T	6-FAM-AGCGCAGCTCCATCATTGCCCTATG-TAMRA
Actin sense-352F	AAGATGACCCAGATCATGTTCGA
Actin antisense-454R	CGATACCAGTGGTACGACCAGA
Actin TaqMan probe-382T	6-FAM-AACACCCCCGCCATGTACGTTGC- TAMRA
18S sense-625F	TCGTAGTTCCGACCGTAAACG
18S antisense-691R	GCCCGGCGGGTCAT
18S TaqMan probe-649T	6-FAM-CCAACTAGCGATCCGGCGG-TAMRA

**Table:** (a) Plasma composition.

	SW	FW
Osmolality (mosmo/kgH_2_O)	302.62 ± 3.32	285.2 ± 3.35**
Mg^2+^ (mmol/liter)	4.7 ± 0.18	2.8 ± 0.35**
Cl^−^ (mmol/liter)	141.4 ± 1.65	124.4 ± 2.21**
Ca^2+^ (free) (mmol/liter)	1.77 ± 0.027	1.8 ± 0.039
Na^+^ (mmol/liter)	151.48 ± 2.39	146.92 ± 2.38
K^+^ (mmol/liter)	2.7 ± 0.038	2.74 ± 0.13

**Table:** (b) Blood composition.

	SW	FW
Hematocrit (Hct) %	25.82 ± 1.3	29.78 ± 1.7
RBC × 10^12^/L	2.258 ± 0.1	2.562 ± 0.2
Hgb g/L	74.4 ± 3.6	84.2 ± 5.4

Independent samples *t*-test was used to assess differences between chronically adapted FW and SW flounder and between experimental and time matched controls at each time point, *P < 0.05, **P < 0.005  (*n* = 7).

## Abbreviations

Hb: Haemoglobin
PCR: Polymerase chain reaction
PCNA: Proliferating cell nuclear antigen
CNSS: Caudal neurosecretory system
NO*_x_*: Nitric oxide and nitrogen dioxide
Hct: Hematocrit.
