# Supplementary material for: Molecular Characterization and Expression of α-Globin and β-Globin Genes in the Euryhaline Flounder (Platichthys flesus)
Source: Evid Based Complement Alternat Med. 2011 Sep 27;2011:965153. doi: 10.1155/2011/965153 (PMC3182402; doi:10.1155/2011/965153)

Supp.FIG.1A

```
                +-------A------+ +-------B------++--C
flounder 1   MSLSGKDKRVVKAIWEKMSSKSDVIGAEALGRMLVSYPQT    40
flounder 2   ----a---sl-rgl-a-aegrvld--g-------------    40
yellowtail A --------s----f-d---p--ae----------tv----    40
yellowtail B ----a---tt--kf-d-veg--ae--g--------a----    40
human 1      -v--pd--tn---a-g-vgahagey-----e--fl-f-t-    40
human 2      -v--pa--tn---a-g-vgahagey-----e--fl-f-t-    40
Functional    B                  B          1  11122
  Residues

             --+          +--------E------+
flounder 1   KTYFSHW.ADLSPSSAPVRKHGATIMAAVGDAVGHMDDLQ    79
flounder 2   ----ae-gt--t-q-qk-gh--gv--g---mg-kyivt-t    80
yellowtail A -------.--vg-d--q-k---------------ki---v    79
yellowtail B -a-----gs--n-qhpm-k----i--ggi-k--kni---v    80
human 1      ----p-f..---hg--q-kg--kkvad-ltn--a-v--mp    78
human 2      ----p-f..---hg--q-kg--kkmad-ltn--a-v--mp    78
Functional   222 2       B                     B
  Residues     HH HH             H  HH

               +---F---+     +----------G------+
flounder 1   GFLSKYSELHAFKLRVDPTNFKILAHNMILVMAMYFPKDF   119
flounder 2   eam-sl------t-----s-------si-------y--e-   120
yellowtail A -g--al------------a--r-----i---t-----t--   119
yellowtail B rg--al------------a--------v---a-----a--   120
human 1      na--al-d---h------v---l-s-cllvtl-ahl-ae-   118
human 2      na--al-d---h------v---l-s-cllvtl-ahl-ae-   118
Functional              B 22 2222     1   1  11B 11 1
  Residues        H  HH   H H   HH  H

             +---------H---------+
flounder 1   TAEVHVSVDKFLQCLALALSEKYR                   143
flounder 2   -------f----s---w-------                   144
yellowtail A -p-i---------n-----a-r--                   143
yellowtail B -p-----------rv----a-r--                   144
human 1      -pa--a-l----asvstv-ts---                   142
human 2      -pa--a-l----asvstv-ts---                   142
Functional   11  11  1             22
  Residues                     H
```

α-globin

SUPP.FIG.1B

```
                 +------A------++-------B------++--C-
flounder 1   MVQWTDSERSAIISLWGKIDVGEIGPQALTRLLIVYPWTQ      40
flounder 2   ----------s--a--------------------------      40
yellowtail A .-d---a--a--s---------------------------      39
yellowtail B --d---a--a--t---------------------------      40
human        --hl-pe-k--vta----vn-d-v-ge--g---v------      40
Functional                                 1  11122
  Residues                                  22

             -+        +--D--++--------E---------+
flounder 1   RHFSTFGDLSTTAAILGSEKVAKHGKTVMGGLERAVKSLD      80
flounder 2   ----------------------------------------      80
yellowtail A ---t---nv--n-----np---q----------n---n--      79
yellowtail B ---t---nv--n-----np---q----------n---n--      80
human        -f-ks------pd-vm-np--ka---k-l-afsdglah--      80
Functional   2  2
  Residues    HH                    H  HH  H

                  +---F---+     +--------G--------+
flounder 1   DIKGVYSALSTMHSEKLHVDPDNFRLLAECISVCVAAKFG     120
flounder 2   ----------------------------------------     120
yellowtail A ---nt-ak--q--------------t--------------     119
yellowtail B ---nt-ak--r--------------a--------------     120
human        nl--tfat--el-cd------e-----gnvlvcvl-hh--     120
Functional                    222 2   2  1   1  11  1
  Residues        H  H  HH   H H   HH  H

                 +---------H---------+
flounder 1   PSVFTAEVQEAWQKFLSVVVSALGRQYH                 148
flounder 2   ----------------------------                 148
yellowtail A kq----d---------------------                 147
yellowtail B kqa---d----------a----------                 148
human        .ke--pp--a-y--vvag-an--ahk--                 147
Functional      1111 11  1             22
  Residues                         H
```

β-globin

Supp.fig.2

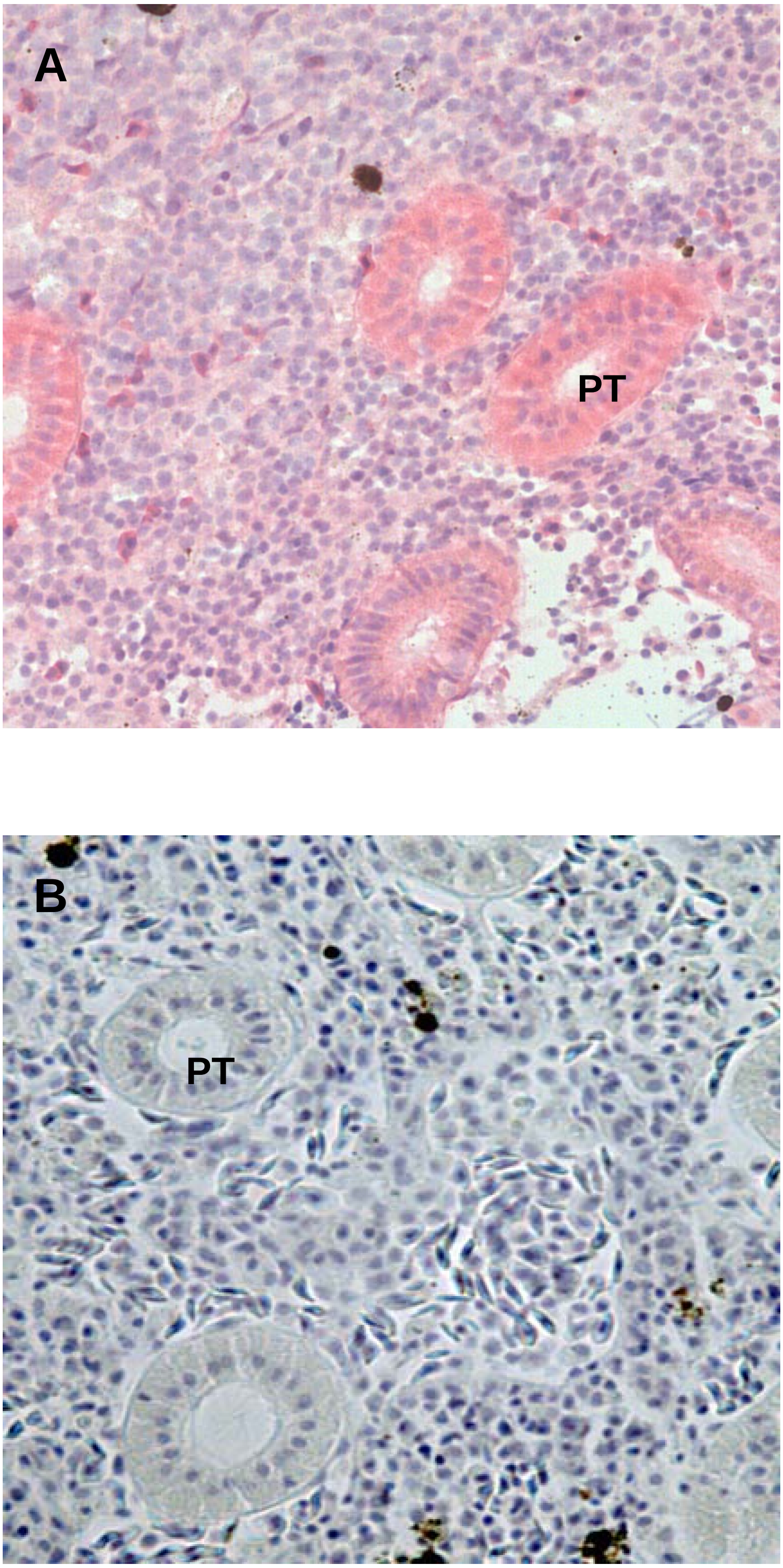

Supplement: Supplementary file 1 — Supplementary Figure 1: Comparison of the predicted amino acid sequences of (a) the flounder β- globin with corresponding chains from previously known α-globin of yellowtail (Okamoto et al., 2001) and human (Michelson and Orkin, 1980) , and (b) the flounder β-globin with β-globin of yellowtail (Okamoto et al., 2001) and human (Lawn et al., 1980). The beginnings and ends of the helical regions, as established for mammalian globins, are indicated by (+) and (-), respectively. The positions of functional residues are indicated by: H, heme binding; 1, α1β1 interfaces; 2, α1β2 interfaces; and B, Bohr effect residues. Supplementary Figure 2: Negative control for figure 5 & 6. (a) In situ hybridization. The same group of cells showed no specific signal when the specific antisence probe for α-globin and β-globin was replaced with sence probe. (b) Immunocytochemistry. The same group of cells showed no immunoreactivity when the specific primary antiserum for α-globin and β-globin was replaced with preimmune rabbit serum. PT, Proximal tubule. [file 965153.f1.pdf]
